# Migraine and Sick Headache

**Published:** 1905-04

**Authors:** 


### Migraine and Sick Headache.

After giving a definition of migraine and sick headache, the author raises the question: "Is there such a thing as idiopathic migraine, or can we assume that where no peripheral cause is apparent, the fault is in our defective means of diagnosis?" He states in reply to this question that there are many sick headaches, which although periodic, occurring in many members of the same family, and having clinical features of a classical attack, can only be differentiated from by a hypothetical true migraine by the fact they follow some obvious peripheral cause, and are relieved when that is removed. But those who suffer in this way are subject to attacks from irritation disproportionately small. Thus, where a patient has sick headache, and is relieved by the correction of half a diopter of astigmatism, the fault is not with the eye primarily, but with the unstable nervous system which made him susceptible, and it is into this that migraine resolves itself. But the fact remains that the cause must be approached at the periphery and the hope of success increases as our diagnostic and therapeutic measures become more perfect.—*Coleman in Medical News.*

